# Characterization of Emerging Pathogens Carrying *bla*_KPC-2_ Gene in IncP-6 Plasmids Isolated From Urban Sewage in Argentina

**DOI:** 10.3389/fcimb.2021.722536

**Published:** 2021-08-24

**Authors:** Barbara Ghiglione, María Sol Haim, Pedro Penzotti, Florencia Brunetti, Gabriela D´Amico González, José Di Conza, Roque Figueroa-Espinosa, Lidia Nuñez, María Tereza Pepe Razzolini, Bruna Fuga, Fernanda Esposito, Maximiliano Vander Horden, Nilton Lincopan, Gabriel Gutkind, Pablo Power, Milena Dropa

**Affiliations:** ^1^Laboratorio de Resistencia Bacteriana, Instituto de Bacteriología y Virología Molecular (IBaViM), Facultad de Farmacia y Bioquímica, Universidad de Buenos Aires, Ciudad Autónoma de Buenos Aires, Argentina; ^2^Consejo Nacional de Investigaciones Científicas y Técnicas, Ciudad Autónoma de Buenos Aires, Argentina; ^3^Facultad de Farmacia y Bioquímica, Cátedra de Salud Pública e Higiene Ambiental, Universidad de Buenos Aires, Ciudad Autónoma de Buenos Aires, Argentina; ^4^Departamento de Saúde Ambiental, Laboratório de Microbiologia Ambiental e Resistência Antimicrobiana - MicroRes, Faculdade de Saúde Pública, Universidade de São Paulo, São Paulo, Brazil; ^5^Departamento de Microbiologia, Instituto de Ciências Biomédicas, Universidade de São Paulo, São Paulo, Brazil; ^6^Ingeniería - Gerencia Técnica, Dirección de Saneamiento, Agua y Saneamientos Argentinos S.A. (AySA), Buenos Aires, Argentina

**Keywords:** *Klebsiella quasipneumoniae*, *Enterobacter asburiae*, KPC-2, IncP-6, wastewater

## Abstract

Untreated wastewater is a reservoir for multidrug-resistant bacteria, but its role in the spread of antibiotic resistance in the human population remains poorly investigated. In this study, we isolated a KPC-2-producing ST2787 *Klebsiella quasipneumoniae* subsp. *quasipneumoniae* (WW14A), recovered from raw sewage at a wastewater treatment plant in Argentina in 2018 and determined its complete genome sequence. Strain WW14A was resistant to all β-lactams, ciprofloxacin and amikacin. A core genome phylogenetic analysis indicated that WW14A was closely related to a GES-5-producing Taiwanese strain isolated from hospital wastewater in 2015 and it was clearly distinct from strains isolated recently in Argentina and Brazil. Interestingly, *bla*_KPC-2_ was harbored by a recently described IncP-6 broad-spectrum plasmid which was sporadically reported worldwide and had never been reported before in Argentina. We investigated the presence of the IncP-6 replicon in isolates obtained from the same sampling and found a novel non-typable/IncP-6 hybrid plasmid in a newly assigned ST1407 *Enterobacter asburiae* (WW19C) also harboring *bla*_KPC-2_. Nanopore sequencing and hybrid assembly of strains WW14A and WW19C revealed that both IncP-6 plasmids shared 72% of coverage (~20 kb), with 99.99% of sequence similarity and each one also presented uniquely combined regions that were derived from other plasmids recently reported in different countries of South America, Asia, and Europe. The region harboring the carbapenem resistance gene (~11 kb) in both plasmids contained a Tn*3* transposon disrupted by a Tn*3*-IS*Apu*-flanked element and the core sequence was composed by ΔIS*Kpn6*/*bla*_KPC-2_/Δ*bla*_TEM-1_/IS*Kpn27*. Both strains also carried genes conferring resistance to heavy metals (e.g., arsenic, mercury, lead, cadmium, copper), pesticides (e.g., glyphosate), disinfectants, and several virulence-related genes, posing a potential pathogenic risk in the case of infections. This is the first study documenting *bla*_KPC-2_ associated with IncP-6 plasmids in *K. quasipneumoniae* and *Enterobacter cloacae* complex from wastewater in Argentina and highlights the circulation of IncP-6 plasmids as potential reservoirs of *bla*_KPC-2_ in the environment.

## Introduction

The spread of carbapenem-resistant Gram-negative bacteria is an urgent and critical public health priority according to the World Health Organization (Tacconelli et al., 2018). Several classes of carbapenemases have emerged in members of the *Enterobacteriaceae* family, including class B metallo-β-lactamases (e.g., New Delhi Metallo-β-lactamase - NDM), class D (e.g., OXA-48) and class A carbapenemases, especially *Klebsiella pneumoniae* carbapenemase (KPC) (Rojas et al., 2017). Antimicrobial resistance genes (ARGs) and their bacterial hosts are widely distributed in clinical settings but also through the environment, especially in surface water, sewage treatment plant effluents, soil, and animal waste (Sekizuka et al., 2018). The appearance of *bla*_KPC_ gene in the environment represents an emerging environmental issue with potentially serious public health implications (Hu et al., 2019). Nevertheless, environmental contamination with carbapenemase-producing *Enterobacteriaceae* (CPE) has not been fully investigated. Compared to clinical isolates, available data for CPE in wastewater are limited, with few reports on genetic characteristics such as fine-scale within-species phylogeny and virulence gene profiles. Detailed characterization of environmental CPE is important to better understand the molecular epidemiology and reservoirs of these clinically important microbes (Gomi et al., 2018). Wastewater Treatment Plants (WWTPs) act as interfaces between the human population and the aquatic environment. Several previous studies have proposed WWTPs and wastewater to be hotspots for horizontal gene transfer, facilitating the exchange of ARGs among different bacterial species. While urban population keeps growing, an increased proportion of sewage effluents get into surface waters, being the role of WWTPs essential in reducing the spread of ARGs. WWTPs and wastewater act as anthropogenic sources, reservoirs, and environmental suppliers of ARGs, making it necessary to monitor the impact on the spread of resistance to antimicrobials (Gomi et al., 2018; Suzuki et al., 2019; Furlan et al., 2020).

Multidrug-resistant hypervirulent lineages of *K. quasipneumoniae* subsp. *quasipenumoniae*, recently defined as a new species, are an emerging issue for public health worldwide. Misidentification using standard laboratory methods is common and consequently, the clinical significance of *K. quasipneumoniae* is imprecisely defined (Rodrigues et al., 2018). While it was originally associated with environmental niches, there is actual evidence that it is able to sustain in hospitalized patients and disseminate among patients (Mathers et al., 2019). Moreover, this species has been shown to take up plasmids from other enterobacteria and harbor several resistance plasmids belonging to different incompatibility groups such as IncU/IncX5 (*bla*_KPC_), IncHI2 (*mcr-9*) and IncFII/IncFIB (*mcr-8.2*) (Mathers et al., 2019; Yang et al., 2019; Faccone et al., 2020).

Members of the *Enterobacter cloacae* complex (ECC) are part of the human gut microbiota and are considered opportunistic pathogens responsible for a wide range of health-care-associated infections and hospital outbreaks, especially in intensive care units. Due to the presence of intrinsic chromosomal AmpC cephalosporinases, along with the acquisition of plasmid-mediated extended-spectrum β-lactamases, carbapenems are among the “antibiotics of choice” to treat infections caused by isolates displaying high-level cephalosporin resistance. Therefore, the increasing carriage of *bla*_KPC_ by members of the ECC over the last years is worrisome (De La Cadena et al., 2018).

Interestingly, KPC-producing *Enterobacteriaceae* and *Aeromonas* isolates from hospital wastewater in Taiwan, river sediments in China or coastal waters in the United States have been recently reported, all of them associated with IncP-6 plasmids (Botts et al., 2017; Gomi et al., 2018; Hu et al., 2019). IncP types are broad-host-range plasmids that have demonstrated the potential to mediate the dissemination of ARGs among Gram-negative bacteria, especially *Enterobacterales* and *Pseudomonas aeruginosa* (Cuzon et al., 2011; Naas et al., 2013; Dai et al., 2016; Gomi et al., 2018).

The KPC-2-encoding gene has been found in several plasmids from different incompatibility (Inc) groups, usually IncFII, IncL/M, IncN, or IncA/C, but only rarely in other plasmids like IncP-6 and IncX (Yao et al., 2017). For example, in a continent-wide study performed in Europe with 1717 K*. pneumoniae* isolates, using a combination of long- and short-read sequence data, *bla*_KPC_-carrying IncP-6 plasmids were found only rarely (David et al., 2020). The emergence of *bla*_KPC_ gene on IncP-6 broad-host-range plasmids, capable of replicating in both *E. coli* (where they are assigned into the IncG group) and *Pseudomonas*, has facilitated its rapid dissemination to *Enterobacteriaceae* and other Gram-negative families (Dai et al., 2016).

The aim of this study was to demonstrate the most relevant genomic features of two KPC-2-producing strains, a *Klebsiella quasipneumoniae* subsp. *quasipneumoniae* and an *Enterobacter asburiae*, isolated from a WWTP in Buenos Aires, Argentina, in 2018. Both strains harbor IncP-6 plasmids, for which we describe the genetic structure, in particular the backbone surrounding *bla*_KPC-2_, and also compare to previously described plasmids, giving an overview of IncP-6 diversity.

This work contributes to a better understanding of acquired resistance and microbial adapting features in *K. quasipneumoniae* and ECC from a One Health perspective and pinpoints the important role of wastewater in global dissemination of resistance markers associated with IncP-6 backbone plasmids across multiple species around the world. To our knowledge, this is also the first report of IncP-6 plasmids circulating in Argentina and supports the hypothesis that IncP-6-*bla*_KPC-2_ promiscuous plasmids may have had a geographic origin in South America prior to their introduction in other countries of Europe and Asia (Yao et al., 2017).

## Materials and Methods

### Sampling and Bacterial Identification

A 1000-mL sample of raw wastewater collected at a municipal sewage treatment plant in Argentina was filtered through 0.45µm membranes, which were cultivated for 18 h at 37°C in MacConkey broth supplied with 10 µg/mL meropenem, to select for bacteria with reduced carbapenem susceptibility. The resulting suspension was then streaked on MacConkey agar and colonies showing different morphologies were selected and assessed by PCR, using primers targeting *bla*_IMP_, *bla*_VIM_, *bla*_NDM_, *bla*_OXA-48_ and *bla*_KPC_ (Poirel et al., 2011). Two *bla*_KPC_ positive strains were isolated and *bla*_KPC-2_ genes were identified in both strains by PCR amplification with primers KPC-F (5´ - ATGTCACTGTATCGCCGTCT - 3´) and KPC-R (5´- TTTTCAGAGCCTTACTGCCC - 3´) (Saba Villarroel et al., 2017) followed by direct amplicon DNA sequencing. Carbapenemase production was checked by a modified Hodge test and a positive result for synergy between imipenem (30 μg) and phenyl boronic acid (300 μg) containing disks. Both isolates were identified to species level by MALDI-TOF/MS (matrix-assisted laser desorption/ionization – time of flight mass spectrometry) (Bruker Daltonics GmbH, Bremen, Germany), and designated WW14A (*Klebsiella pneumoniae*) and WW19C (*E. asburiae*).

### Antimicrobial Susceptibility Testing

Antibiotic susceptibility testing was performed by disk diffusion for 14 antimicrobial compounds: ampicillin (10 µg), amoxicillin/clavulanic acid (20/10 µg), cefazolin (30 µg), cefoxitin (30 µg), ceftriaxone (30 µg), ceftazidime (30 µg), aztreonam (30 µg), meropenem (10 µg), gentamicin (10 µg), amikacin (30 µg), ciprofloxacin (5 µg), trimethoprim/sulfamethoxazole (23.75/1.25 µg), chloramphenicol (30 µg) and tetracycline (30 µg). Colistin susceptibility was assessed by broth dilution, while minimal inhibitory concentration (MIC) of the following β-lactam antibiotics was carried out by agar dilution according to CLSI 2021 guidelines (CLSI, 2021): ampicillin, piperacillin, piperacillin/tazobactam, cephalothin, cephalexin, cefoxitin, ceftriaxone, ceftazidime, cefepime, imipenem and meropenem.

### Mobilization Experiments

The transmissibility of plasmids was tested with the filter mating protocol using *E. coli* J53 (sodium azide resistant) as recipient. Mating was initiated by mixing 0.5 mL donor bacteria suspension with 0.5 mL recipient bacteria (16 h culture in lysogeny broth; LB) followed by immobilization onto a 0.22-µm nitrocellulose membrane filter. Control cultures were prepared identically with either donor or recipient bacteria alone. Filters were incubated overnight at 35°C on LB agar plates (no antibiotics). Biomass was removed with a sterile cotton swab and re-suspended in 1 mL sterile saline. Fifty µL of suspended bacteria, either donor, recipient, or the mating combination, were spread onto LB agar plates supplemented with 50 µg/mL sodium azide and 10 µg/mL imipenem and incubated at 37°C for 18 h.

### Short-Read Whole Genome Sequencing and Analysis

For whole genome sequencing (WGS) analyses, strains WW14A and WW19C were streaked to single colonies on MacConkey agar plates containing 2 µg/ml imipenem and then grown for 18 h at 37°C in 3 mL of LB. Total genomic DNA was extracted using PureLink quick gel extraction kit (Life Technologies, CA). DNA was quantified using the Qubit dsDNA HS assay system (Life Technologies, CA). Genomic library was constructed using the Nextera DNA Flex library preparation kit (Illumina, San Diego, CA) and, subsequently, sequenced on an Illumina MiSeq platform to generate 2 × 250 base paired-end reads. Sequenced reads were *de novo* assembled using SPAdes (v3.11.1) (Bankevich et al., 2012). Automated annotation was performed using PROKKA (1.14.6) (Seemann, 2014) and RAST server (https://rast.nmpdr.org/). Species identification was confirmed from assemblies by ANI analysis (http://enve-omics.ce.gatech.edu/ani/) and using the free online resource Pathogenwatch (https://pathogen.watch/). STs were defined by submitting genome sequences to official MLST databases for *Klebsiella pneumoniae* (https://bigsdb.pasteur.fr/klebsiella/klebsiella.html) and ECC (https://pubmlst.org/organisms/enterobacter-cloacae).

Capsular type for WW14A was assessed using BIGSdb (https://pubmlst.org/software/bigsdb) and KAPTIVE (https://kaptive-web.erc.monash.edu/) databases. Plasmid types and incompatibility (Inc) groups were defined based on their replicon genes (*rep*) using the PlasmidFinder database available at the Center for Genomic Epidemiology (CGE) (http://www.genomicepidemiology.org/). Other databases available at the CGE were also used to seek antimicrobial resistance determinants (ResFinder), virulence genes (VirulenceFinder) and mobile genetic elements (MGE). Prophages sequences were identified and annotated using the web server PHASTER (https://phaster.ca/), while the detection of CRISPR signatures was carried out using the online tool CRISPRfinder (https://crisprcas.i2bc.paris-saclay.fr/) (Grissa et al., 2007; Couvin et al., 2018).

### Pangenome Analysis

Pangenome analysis of WW14A and WW19C was performed for constructing whole genome single nucleotide polymorphism (SNP)-based within-genus phylogenetic trees, selecting isolates analyzed in other studies (**Supplementary Table S1**), i.e., isolates analyzed by Chavda et al. (2016), Gomi et al. (2018) and Jousset et al. (2019) for WW19C; and isolates analyzed by Gomi et al. (2018) and all *Klebsiella quasipneumoniae* genomes present in NCBI Assembly database (June 5^th^, 2020) for WW14A, aiming to place WW19C and WW14A in broader phylogenetic contexts. Briefly, annotated genomes were used in a pangenome analysis with Roary 3.13.0 to generate a core-gene alignment (using a blastp percentage identity of 95% and a core definition of 99%) (Page et al., 2015). This core-genome alignment was used to generate a SNP alignment with SNP-sites (v 2.5.1) (Page et al., 2016) which was later used to construct a maximum likelihood (ML) phylogenetic tree with RAxML (v 8.2.12) (Stamatakis, 2014) under the generalized time reversible model (GTR) and 100 bootstrap replicates.

### Long-Read WGS and Plasmids Analysis

Nanopore sequencing was performed according to the manufacturer’s instructions. Briefly, DNA libraries were prepared using a rapid sequencing kit (Oxford Nanopore Technologies), and the prepared library was subsequently loaded into a MinION flow cell (R9.4; Oxford Nanopore Technologies). Raw data (FAST5 files) were base-called, converted to FASTQ format and trimmed of barcode and adapter sequences using Guppy v3.0.3 (Ueno et al., 2003). Hybrid assemblies of both short and long reads were performed using Unicycler v0.4.8 (Wick et al., 2017) and annotated with Prokka (1.14.6) (Seemann, 2014) and RAST server (https://rast.nmpdr.org/). Manual annotation of IncP-6 plasmids was additionally performed using National Center for Biotechnology Information (NCBI) BLASTn (Altschul et al., 1990) and Uniprot databases (Consortium, 2019). The annotation of ISs was performed using ISFinder (https://isfinder.biotoul.fr/) (Siguier et al., 2006). Final hybrid assemblies were compared to publicly available IncP-6 plasmids using BRIG (Alikhan et al., 2011), and comparison of plasmids was facilitated using the Artemis Comparison Tool (Carver et al., 2005).

## Results and Discussion

Membrane filtration and cultivation using meropenem selection of the wastewater sample followed by PCR screening allowed the detection of CPE, from which two KPC-producing strains, WW14A and WW19C, were selected for further analysis and whole genome sequencing.

**Tables 1** and **2** summarize the genomic features observed in each strain, based on annotation outputs. The most relevant results of this analysis will be detailed and discussed in the following sections.

**Table 1:** Genome Features observed in multidrug-resistant wastewater isolates WW14A and WW19C.

	*Klebsiella quasipneumoniae* subsp. *quasipneumoniae* WW14A	*Enterobacter asburiae* WW19C
**Sequence type/Capsular type**	2787/close to wzi88-KL70	1407 (new)/-
**Plasmids and Phages**
Incompatibility Groups	IncP-6, IncFIB, IncFII, IncR IncHI1	IncP-6, IncFIB(pECLA), IncHI2
Non-typeable	113kb; 8.8kb; 3.5kb; 2.9kb	4.9kb; 4.1kb; 3.9kb; 3.1kb; 1.3kb
Complete Prophage sequences	*Haemophilus* phage SuMu (NC_019455)	*Escherichia* phage HK75 (NC_016160)
		*Salmonella* phage SEN34 (NC_028699)
		*Escherichia* phage 186 (NC_001317)
		*Enterobacter* phage Tyrion (NC_031077)
**Virulence and Maintenance genes**
***Toxins***		
Aerobactins	*iutA-fhuABCDF*	*iucABCD*, *iutA-fhuABCDF*
Enterobactins	*entABCDEFHS*, *fepABCDG*, *ybdZ*	*entABCDEFHS*, *fepABCDEG*, *ybdZ*
Hemolysins	*hha/tomB*, *hlyB* (T1SS), *shlB*	*hha/tomB*, *hlyBD* (T1SS), *shlB1,2,3,4*
Pullulanase secretion	*pulADGS*	*pulG*
***Motility and adhesion***		
Type 1 fimbriae	*fimABDEFGHI*, *staA*, *ecpABCDER*, *mrkD*, *elfA*, *yadNV*, *smf-1*	*fimABDEFGHIZ*, *sfmACDFH*, *staA*, *sfaS*, *yadN*, *yraI*, *ycbV*, *mrpA, elfD*, *prsE*
Type 4 fimbriae	*pilABCMNOPQT*	*pilABCMNOPQT*
Long polar fimbriae	*lpfAB*	*lpfAB*
Flagella	*flk*, *ycgR*	*flgABCDEFGHIKLMN*, *fliCDEFGHIJKLMNOPQRST*, *flhABCDE*, *flk*, *motAB*, *ycgR*
Biofilm formation	*csgD*, *pgaABCD*, *bssS*, *bhsA*, *bdcA*, *bigR*	*csgABCDEFG*, *pgaABCD*, *bssS*, *bhsA*, *bdcA*
Cell invasion	–	*sctC* (T3SS)*, tssABCJKLM* (T6SS)
***Defense, growth and adaptation***		
Export of proteins	T2SS: *gspFHK*, *outCN*, *epsEFLM*, *xcpW*; T4SS: *outO*	T2SS: *gspDEFGHL*, *outC*, *epsE*; T4SS: *outO*
Osmotic shock defense	–	*ybdG*
Uptake and transport systems	Iron: *efeBOU*, *feuC*, *fetAB*, *iscRSUAX*	Iron: *efeBOU*, *feuC*, *fetAB*, *iscRSUAX*
	Zinc: *znuABC*, *zupT*, *zur*	Zinc: *znuABC*, *zupT*, *zur*
	Glycine: *yehWXYZ*, *proVWX*	Glycine: *yehWXYZ*, *proVWX*
Toxin-antitoxin systems	*higAB*, *higA1*, *hipAB*, *tabA*, *yfjZ/ypjF*, *cbtA/cbeA*, *ccdAB*, *relBE*, *mazEG*, *yefM*	*higA*, *higB-1*, *higB2*, *hipA*, *pasIT*, *parD1*, *relB*, *tabA*, *yfjZ*, *ykfI*
General regulators	*dsbABCDG*	*dsbABCDG*

**Table 2:** Antimicrobials, heavy metals and organic compounds resistance genes identified in WW14A and WW19C genomes.

		*K. quasipneumoniae* subsp. *quasipneumoniae* WW14A	*E. asburiae* WW19C
Antimicrobials	Aminoglycosides	*aac(6’)-Ib-cr*, *aadA1*	*aac (6’)-Ib-cr*, *aadA1*
	Beta-lactams	*bla*_KPC-2_, *bla*_OXA-9_, *bla*_TEM-1A_	*bla*_KPC-2_, *bla*_ACT-57_, *bla*_OXA-9_, *bla*_TEM-1A-like_
	Chloramphenicol	–	*catA1*
	Fluoroquinolones	*gyrAB* and *parCE* mutations, *aac(6’)-Ib-cr*, *oqxAB*-like (94 and 95%) (efflux)	*qnrB10*, *aac (6’)-Ib-cr*, *oqxAB*-like (86 and 89%) (efflux)
	Fosfomycins	*fosA* (chromosomal)	*fosA/fosA2* (chromosomal), *abaF* (efflux)
	Fusidic acid	*fusBCDE*	–
	Macrolides	macAB (efflux)	*ereA*, *macAB* (efflux)
	Polymyxins	*emrA* (efflux), *phoP* mutation	*emrA* (efflux)
	Sulfonamides/Trimethoprim	–	*sul1*, *dfrA8*
Heavy Metals	Arsenic	*arsBCR* (efflux)	*arsBCHR* (efflux)
	Cadmium/Zinc/Cobalt	*zitB*, *czcABC*	*zitB*, *czcABC*
	Chromium	*chrAB*	*chrAB*
	Copper	*copCD*, *cueOR*, *cpxAR/scsABCD*	*copABCDG*, *pcoD*, *cueOR*, *cpxAR/scsABCD*
	Copper/Silver	*cusABCFRS* (efflux)	*cusABCFRS* (efflux)
	Magnesium/cobalt/nickel/manganese	*mgtAE*, *corAC* (efflux)	*mgtAE*, *corAC* (efflux)
	Manganese	*mntHPR* (efflux)	*mntHPR* (efflux)
	Mercury	*merPRT*	*merCDEPRT*
	Nickel	*nikABCDER*	–
	Nickel/Cobalt	*cnrA*, *STM2551* (efflux), *rcnABR* (efflux)	*cnrA*, *STM2551* (efflux), *rcnABR* (efflux)
	Silver	*silA* (variant)	–
	Tellurium	*tehAB*	*terABCD*, *tehAB*
Organic Compounds	Atrazine	*atzE*	*atzE*
	Glyphosate	*phnABCDEFGHIJKLMNPRSTUVWX*	*phnCDEFGHIJKLMNOP*
	Organic hydroperoxide	–	*ohrB*
	Quaternary ammonium compounds	*qacC*, *sugE*	*qacE*
	Toluene	–	*ttgEFG*
	Triclosan	*fabG* and *gyrA* mutations	–
Multiple compounds (Efflux Systems)		*acrABDEFRZ*, *mdtABCGHKLMNOPQ, mdfA/cmr*, *emrABDR*, *marAR*, *norM*, *soxSR*, *ramAR*, *fis*	*acrAB*, *mdtABCDHKLQ*, *mdfA/cmr*, *emrAB, marABCR*, *mexAB*

### *Klebsiella quasipneumoniae* subsp. *quasipneumoniae* WW14A

Strain WW14A was initially identified as *Klebsiella pneumoniae* by MALDI-TOF/MS. *K. pneumoniae* (phylogroup Kp1/KpI) is phylogenetically related to *K. quasipneumoniae* [subsp. *quasipneumoniae* (Kp2/KpIIA) and subsp. *similipneumoniae* (Kp4/KpIIB)], *K. variicola* (Kp3/KpIII) and two unnamed phylogroups (Kp5 and Kp6). Together, Kp1 to Kp6 make-up the *K. pneumoniae* complex. The phylogroups can be reliably identified based on genome sequencing but only recently Rodrigues et al. demonstrated the potential of MALDI-TOF/MS for precise identification of *K. pneumoniae* complex members. Incorporation of spectra of all *K. pneumoniae* complex members into reference MALDI-TOF/MS spectra databases, in which they are currently lacking, is desirable (Rodrigues et al., 2018).

**Figure 1:**
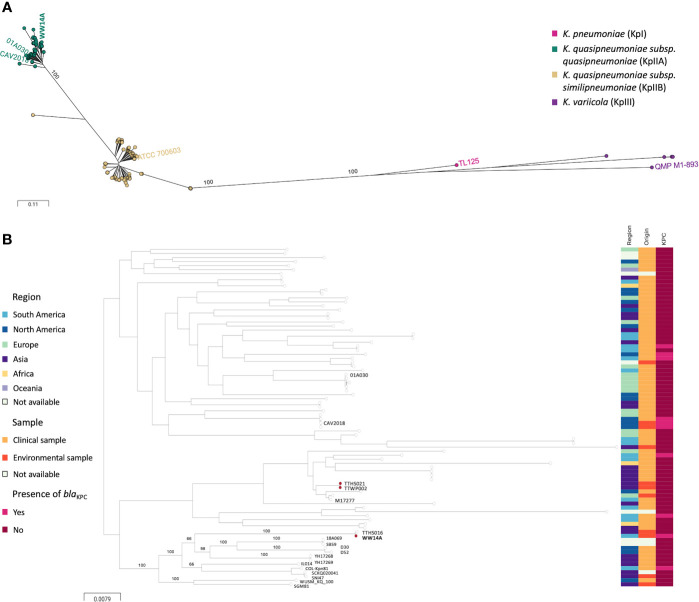
Phylogenetic analysis of strain WW14A. **(A)** ANI (Average Nucleotide Identity) analysis was performed using JSpeciesWS. *Klebsiella quasipneumoniae* subsp. *quasipneumoniae* was confirmed by constructing whole genome single nucleotide polymorphism (SNP)-based within-species/genus phylogenetic tree. **(B)** Core genome phylogenetic analysis of strain WW14A. The analysis showed that strain WW14A is closely related to a Taiwanese GES-5-producing *Klebsiella quasipneumoniae* subsp. *quasipenumoniae* isolate from hospital wastewater (TTHS016) and is clearly distinct from strains isolated recently from Argentina (M17277; Faccone et al., 2020) and America. TTHS021, TTWP002 and WW14A strains are marked with a pink dot, indicating they carry an incP-6 plasmid. (*Klebsiella quasipneumoniae* genomes present in NCBI Assembly database were used for comparison. Data was graphically displayed using Microreact at microreact.org).

Sequence analysis revealed that WW14A isolate includes a genome of 6.01 Mb with 57.3% GC content. It was confirmed as *Klebsiella quasipneumoniae* subsp. *quasipneumoniae* and assigned as ST2787. Moreover, a core genome phylogenetic analysis indicated that strain WW14A is closely related to a GES-5 producing *Klebsiella quasipneumoniae subsp quasipenumoniae* isolate from a Taiwanese hospital wastewater (Gomi et al., 2018) and is clearly distinct from strains isolated recently in Argentina (Faccone et al., 2020) and Brazil (Fuga et al., 2020; Furlan et al., 2020) (**Figure 1**).

According to BIGSdb and KAPTIVE databases, the closest capsular type assigned for this strain is wzi88-KL70, considering there are 2 mismatches with the reference *wzi* cluster 88 and 78.7% identity with KL70 locus (9 from 20 genes present). The strain displayed a negative string test, so it may not be classified as hypermucoviscous. On the other hand, virulome analysis showed a hypervirulent profile, carrying genes encoding for aerobactins, enterobactins, hemolysins, pullulanase secretion, hyperadherence and biofilm formation. One intact phage-related sequence (*Haemophilus* phage SuMu [39.8 kb]) was identified, besides other ten incomplete (7.2 to 34.7 kb) or questionable phages from *Pseudomonas*, *Salmonella*, *E. coli* and *Cronobacter*. Also, two confirmed (178 and 459 bp) and four questionable (94 to 121 bp) CRISPRs signatures were identified. The presence of phages and CRISPRs in WW14A genome shows its adaptative signatures, i.e., a memory of past genetic interactions with bacteriophages and plasmids (**Table 1**).

Resistome analysis showed antimicrobial resistance encoded by chromosomal mutations and by acquired resistance genes for fluoroquinolones, aminoglycosides, sulfonamides, trimethoprim and polymyxins. TEM-1A, OXA-9 and KPC-2 β-lactamases were present. Also, WW14A carried resistance genes for several metals (arsenic, cadmium, cobalt, magnesium, manganese, mercury, nickel, silver) and organic compounds like detergents and pesticides (**Table 2**).

WW14A was resistant to all tested β-lactams, ciprofloxacin, amikacin, and trimethoprim-sulfamethoxazole but susceptible to gentamicin, tetracycline, chloramphenicol, and colistin. β-lactam MICs showed the strain was highly resistant to ceftazidime, ceftriaxone, imipenem, meropenem, piperacillin/tazobactam, ampicillin, piperacillin, cephalothin, cefalexin and cefoxitin (**Table 3**).

**Table 3:** β-lactam MIC values for WW14A and WW19C strains.

Antibiotic	MIC (µg/mL)	
	WW14A	WW19C
Ampicillin	>256	>256
Piperacillin	>256	>256
Piperacillin/tazobactam	>256	>256
Cephalothin	>256	>256
Cephalexin	>256	>256
Cefoxitin	128	>256
Ceftriaxone	32	32
Ceftazidime	32	32
Cefepime	32	8
Imipenem	128	64
Meropenem	>256	128

Seven plasmids were detected, but only five plasmid incompatibility groups could be assigned by PlasmidFinder: pWW14A-IncFII/IncR (228495 bp), pWW14A-3 (untypable; 113444 bp), pWW14A-IncFIB/IncHI1B (96771 bp), pWW14A-KPC2 (IncP6; 40407 bp), pWW14A-6 (untypable; 8819 bp), pWW14A-7 (untypable; 3478 bp) and pWW14A-8 (untypable; 2954 bp). IncFII(K) contained the Tra conjugative system, while IncP-6 harbored the KPC-2 transposon.

WW14A is the first non-clinical KPC-2-producing isolate from Argentina which has been fully sequenced. *Klebsiella quasipneumoniae* was recently defined as a new species and was originally thought to be associated exclusively with environmental niches (Venkitapathi et al., 2021). However, despite there being relatively few reports to date, the true prevalence of this organism in clinical settings is likely underestimated as it is not generally distinguished from *K. pneumoniae* in routine testing of clinical laboratories (Mathers et al., 2019).

### *Enterobacter asburiae* WW19C

**Figure 2:**
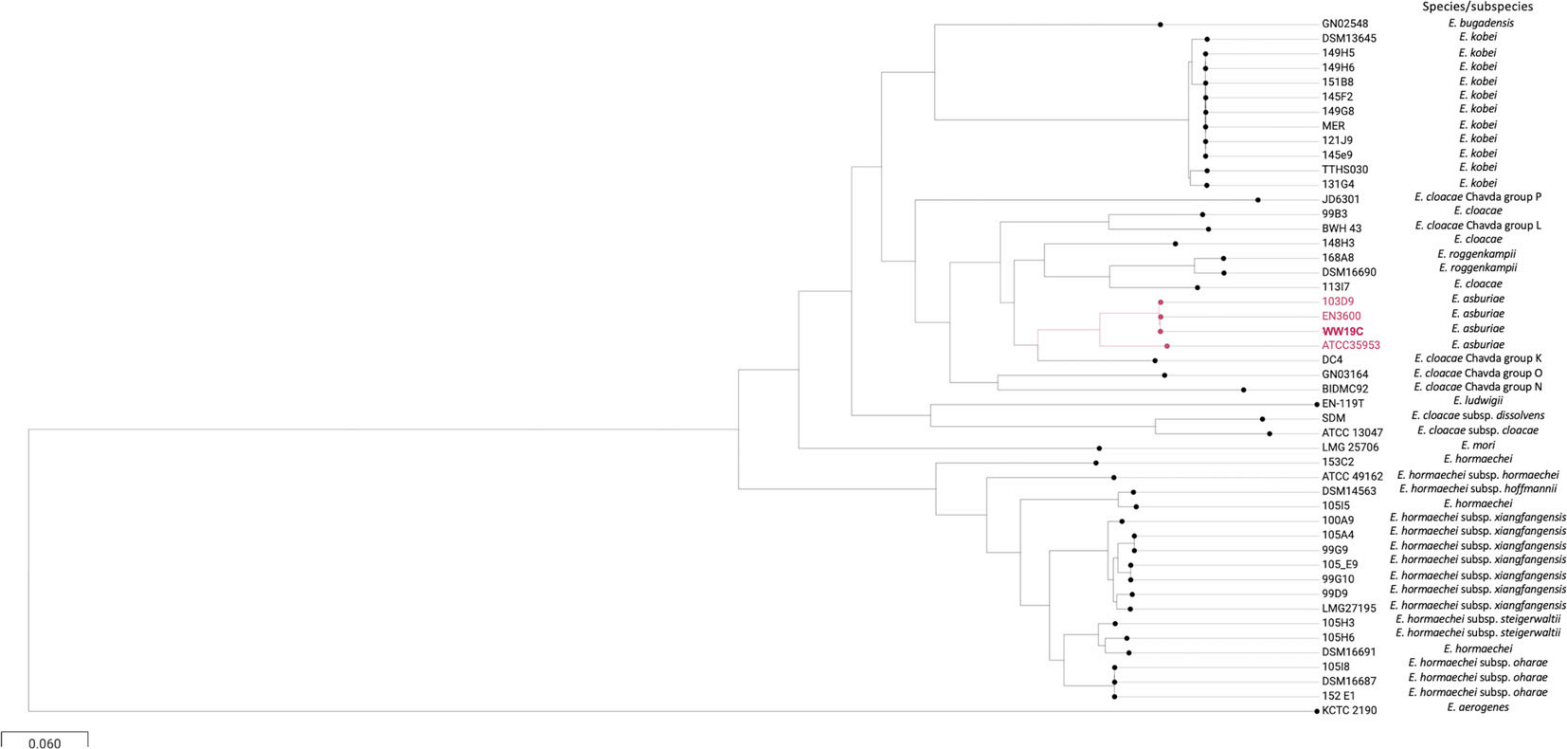
Phylogenetic analysis of WW19C. ANI (Average Nucleotide Identity) analysis was performed using JSpeciesWS. It was confirmed by constructing whole genome single nucleotide polymorphism (SNP)-based within-species/genus phylogenetic trees.

WW19C was identified as *E. asburiae* with a 5.2 Mb genome and 55.2% GC content. The phylogenomic analysis confirmed that WW19C was related to *E. asburiae* EN3600, a clinical isolate co-producing IMP-8, CTX-M-14, CTX-M-3, and QnrS1 from China (**Figure 2**) (Yuan et al., 2019). MLST analysis revealed a new allele for *fusA* gene (allele 245), which associated with other alleles (*dnaA* [24], *gyrB* [43], *leuS* [52], *pyrG* [27], *rplB* [18], and *rpoB* [21]) generated the new ST1407.

Regarding its virulome, when compared to WW14A, WW19C shows the same categories of virulence, but with a broader variety of genes. Four intact phage-related sequences were identified: *Enterobacter* phage Tyrion (46.4 kb), *Escherichia* phages HK75 (34 kb) and 186 (26 kb), and *Salmonella* phage SEN34 (42.5 kb), the last being also found as an incomplete phage sequence in WW14A. In addition, WW19C also carried two incomplete (6.8 kb and 28.1kb) and two questionable phage sequences. Three questionable CRISPRs (96, 134 and 148 bp) were also identified (**Table 1**).

Resistome analysis showed ARGs for fluoroquinolones, aminoglycosides, sulfonamides, trimethoprim, macrolides and polymyxins. Chromosomal β-lactamases TEM-1-like (A213T variant) and AmpC ACT-57, and plasmid-borne KPC-2 were present. Also, several metal resistance genes, efflux systems and organic compounds resistance genes could be identified, as detailed in **Table 2**.

WW19C expressed a MDR phenotype, showing susceptibility to tetracycline, intermediate susceptibility to amikacin and resistance to all tested β-lactams, ciprofloxacin, gentamicin, trimethoprim-sulfamethoxazole, chloramphenicol and colistin. β-lactam MICs showed the strain was highly resistant to ceftazidime, ceftriaxone, imipenem, meropenem, piperacillin/tazobactam, ampicillin, piperacillin, cephalothin, cefalexin and cefoxitin (**Table 3**).

Eight plasmids were detected but only four incompatibility groups could be assigned by PlasmidFinder: pWW19C-IncHI2 (332184 bp), pWW19C-IncFIB (139140 bp), pWW19C-KPC2 (IncP6; 34721 bp), pWW19C-5 (untypable; 4935 bp), pWW19C-6 (untypable; 4152 bp), pWW19C-7 (untypable; 3872 bp), pWW19C-8 (untypable; 3108 bp), pWW19C-9 (untypable; 1282 bp).

Members of the ECC are opportunistic pathogens responsible for a wide range of healthcare-associated infections and hospital outbreaks, especially in intensive care units (De La Cadena et al., 2018). The emergence of carbapenem-resistant ECC like WW19C is of great concern as diverse plasmids of incompatibility group IncP-6 (20 – 60 kb) were the most common carriers of *bla*_KPC_ among ECC clinical isolates from Colombia, suggesting that KPC dissemination is not just due to horizontal transmission of a single plasmid-borne *bla*_KPC_, but that multiple rearrangements and transposition events can occur (Rojas Coy et al., 2019).

### Overview of IncP-6 Plasmids Carrying *bla*_KPC-2_

**Figure 3:**
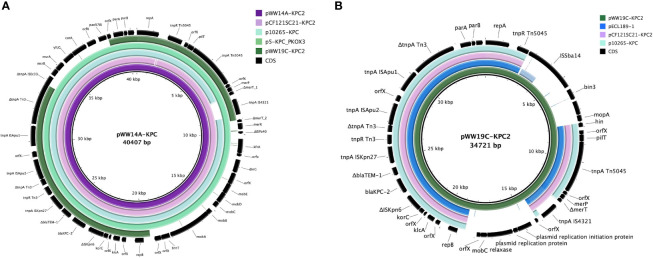
Comparison of pWW14A-KPC2 and pWW19C-KPC2 with IncP-6 plasmids available in public databases. **(A)** Comparisons with pWW14A-KPC2. Analysis showed that the structure was highly similar to those of several *bla*_KPC-2_ -containing plasmids of both environmental and clinical origin deposited in GenBank. One plasmid p5-KPC_PKOX3 from clinical *Koxytoca* (GenBank accession no. KY913901, 100% query coverage and 99,93% nucleotide identity), one plasmid p121SC21-KPC2 from wastewater *C. freundii* (Genbank accession no. NZ_LT992437.1, 99% query coverage and 99,93% nucleotide identity) and p10265-KPC from clinical *P. aeruginosa* 10265 (GenBank accession no. KU578314, 96% query coverage and 99,78% nucleotide identity). **(B)** Comparisons with pWW19C-KPC2. This plasmid shows the highest similarity with pECL189-1 from *E. hormaechei* ECL189 (GenBank accession no. CP047966.1, 72% coverage and 100% identity), a strain isolated from a Chinese hospital co-producing KPC-2, NDM-1, TEM-1 and SHV-66 (data submitted to GenBank on 15 January 2020, unpublished).

IncP-6 plasmids identified in this study, designated pWW14A-KPC2 and pWW19C-KPC2, could be completely closed, analyzed, and compared with other closed plasmids belonging to the same incompatibility group. They both share 72% of coverage, with 99.99% of sequence similarity. **Figure 3** shows a comparison of IncP-6 plasmids described herein with other complete IncP-6 described in the literature (Naas et al., 2013; Dai et al., 2016; Wang et al., 2017; Yao et al., 2017).

The *bla*_KPC-2_-containing plasmid pWW14A-KPC2 was 40,407 bp in size with an average GC content of 57.9%. It comprised 55 open reading frames (ORF) according to RAST annotation, of which 25 encoded proteins with known functions and 30 were hypothetical proteins. Further analysis showed that the structure was highly similar to those of several *bla*_KPC-2_-containing plasmids of both environmental and clinical origin deposited in GenBank. Plasmids pKOX3-P5-KPC, from a clinical *K. oxytoca* in China (GenBank accession no. KY913901) (Wang et al., 2017), p121SC21-KPC2 from Spanish wastewater *Citrobacter freundii* 121SC21 (Genbank accession no. NZ_LT992437.1) (Yao et al., 2017), and p10265-KPC, first reported in China from a clinical *P. aeruginosa* 10265 (GenBank accession no. KU578314) (Dai et al., 2016) were highly similar over the entire region (**Figure 3**). The *bla*_KPC-2_ gene is the only determinant of antimicrobial resistance located in plasmid pWW14A-KPC2. This is the first report of a typical IncP-6 plasmid carrying *bla*_KPC-2_ in a *K. quasipneumoniae* isolate.

On the other hand, pWW19C-KPC2 was 34,721 bp with an average GC content of 52.9%. It comprised 46 open reading frames (ORF) according to RAST annotation, of which 25 encoded proteins with known functions and 21 were hypothetical proteins. It showed the highest similarity with pECL189-1 from *Enterobacter hormaechei* ECL189 (GenBank accession no. CP047966.1, 72% coverage and 100% identity), a strain isolated from a Chinese hospital co-producing KPC-2, NDM-1, TEM-1 and SHV-66 (**Figure 3**).

### Replication, Maintenance and Dissemination of pWW14A-KPC2 and pWW19C-KPC2

Both plasmids belong to the IncP-6 incompatibility group according to replicon-based schemes because they carry the replicase gene *repA*, which constitutes an IncP-6-type consecutive *par-rep* gene cluster, together with the partition genes *parABC*. The *repA* gene and the *parABC* locus of pWW14A-KPC2 and pWW19C-KPC2 are identical and show 100%, >96%, >98%, and >98% of nucleotide sequence identity with the IncP-6 plasmids p10265-KPC, Rms149, pRIO-5, and pCOL-1, respectively. RepA in Rms149 has shown to confer the plasmid’s replication ability in *E. coli*, *P. aeruginosa*, and *P. putida* and its *parABC* locus is known to promote plasmid mobilization in *E. coli* (Haines et al., 2005), while RepA in pRIO-5 allows replication in *Serratia marcescens* and *Acinetobacter baumannii* but not in *P. aeruginosa* (Bonnin et al., 2012). pWW19C-KPC2 also contains and extra plasmid replication initiation protein with 100% nucleotide identity with the replicon of pVPS18EC0801-5, a short non-typeable plasmid of 4910 bp present in foodborne *E. coli* strain 18GA07VL07-EC isolated from retail veal in the United States in 2018 (GenBank accession no. CP063722.1, unpublished). The presence of two replication initiation genes, IncP-6 replicon *rep*A and the replication initiation protein found in pVPS18EC0801-5 suggests pWW19C-KPC2 may be a novel hybrid plasmid (**Supplementary Figure S1**). The presence of extra replicon genes may expand pWW19C-KPC2 ability of maintenance and dissemination.

**Figure 4:**
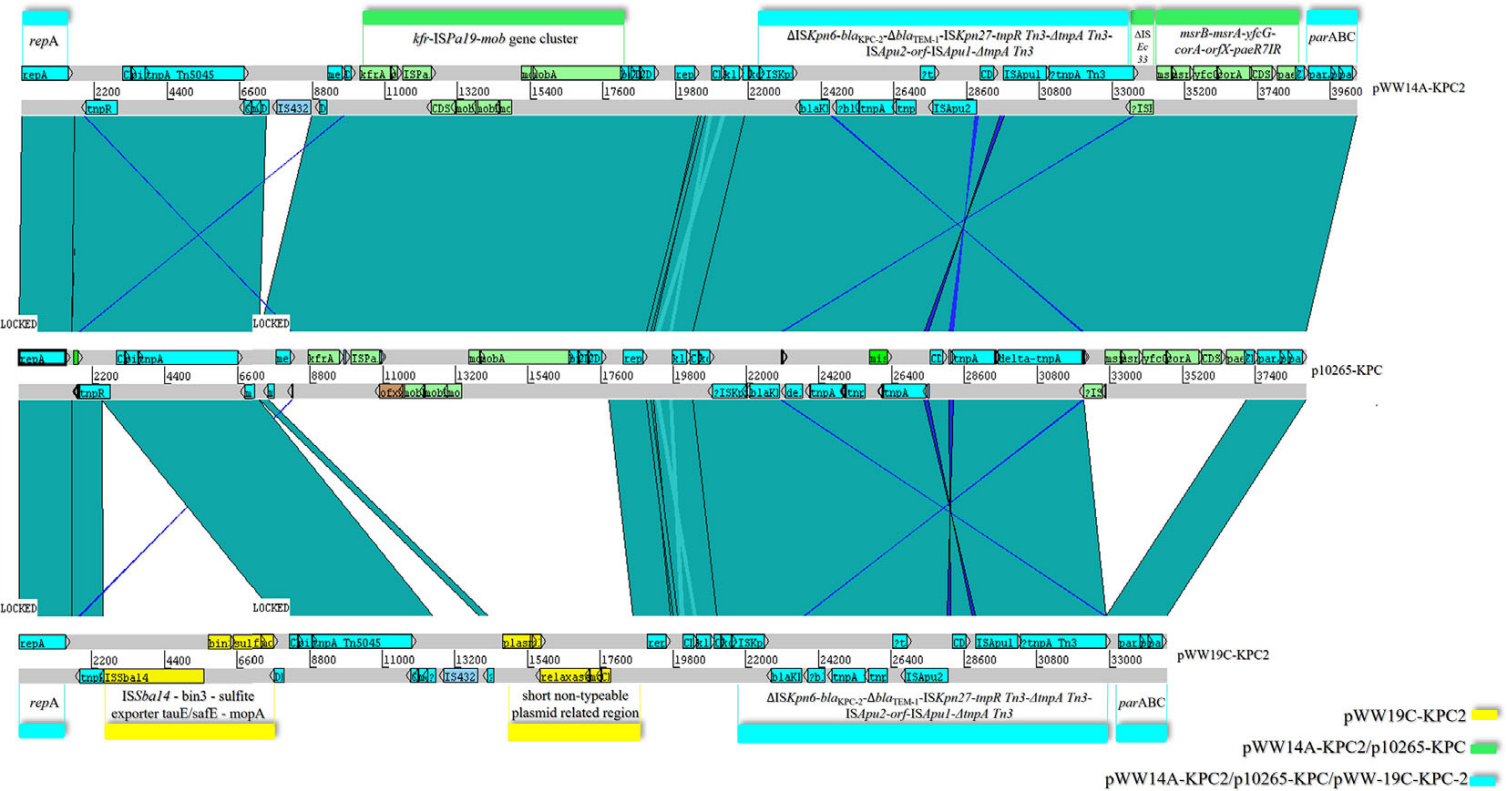
Linear comparison of p10265-KPC with pWW14A-KPC2 and pWW19C-KPC2. Turquoise regions denote shared regions of homology (>95% nucleotide similarity). Some regions are denoted in detail, in cyan for gene clusters shared by pWW14A-KPC2/p10265-KPC/pWW19C-KPC2, in green for gene clusters shared by pWW14A-KPC2/p10265-KPC and in yellow for regions exclusively present in pWW19C-KPC2. Figure generated using Artemis Comparison Tool (ACT).

The IncP-6 backbone of pWW14A-KPC2 was compared with p10265-KPC and it contained the same plasmid maintenance genes, as follows: *kfrA*; a 5.6 kb MOBP family mobilization module composed of genes *mobA* (relaxase/primase fusion protein), *mobB* (oriT recognition-like protein), *mobC* (relaxosome protein), *mobD* and *mobE* (auxiliary proteins); the anti-oxidative system *msrB-msrA-yfcG-corA-orfX* gene cluster; and endonuclease paeR7IR. Within the backbone, pWW14A-KPC2 harbored two accessory modules: on one hand, a truncated Tn*5045*-associated mercury resistance operon disrupted by IS*4321* (the latter being absent in p10265-KPC), and on the other, IS*Pa19*. Linear comparison of the above-mentioned plasmids with pWW19C-KPC2 showed notable differences (**Figure 4**):

- Absence of a ~10 kb region that includes *kfrA*, IS*Pa19* and the MOBP family mobilization module;
- Absence of a ~ 4.9 kb region that includes the anti-oxidative system and paeR7IR;
- The number of copies of the 17 bp oriV iteron sequence (GCGCCTGCCTTTGAGTA) was 6 in p10265-KPC and 14 in pWW14A-KPC2, in contrast to pWW19C-KPC2 where it appeared only 8 times;
- The presence of a ~5.2 kb extra region composed by the cluster IS*Sba14*-Tn*552* invertase bin3-transmembrane sulfite exporter tauE/safE - mopA. Blast search indicated that the extra region is present in other four plasmids of diverse origins with more than 99.9% identity (**Supplementary Table S2**), and it is also completely shared by the IncFI2 plasmid also present in *E. asburiae* WW19C.

None of the plasmids could be transferred to *E. coli* J53 *via* conjugation, despite repeated attempts. The examination of plasmid sequences confirmed the absence of conjugative transfer genes involved in plasmid transfer, such as the *tra* or *trb* operons, in accordance with the fact that typical IncP-6 plasmids are mobilizable rather than self-transmissible if a conjugative plasmid is also present (Botts et al., 2017). The *mob* gene cluster in pWW14A-KPC2 is functional for plasmid mobilization in *E. coli* (Yuan et al., 2019), being *mobA*, *mobB*, and *mobC* essential for functionality while *mobD* and *mobE* are non-essential but greatly enhance mobilization frequency (Dai et al., 2016). In the case of pWW19C-KPC2, which lacks this module, we inferred that this function is assumed by the relaxosome proteins coded in the unique accessory region related to the short non-typeable plasmid pVPS18EC0801-5 (**Figure 4**).

While IncP-6 plasmids are naturally isolated from *P. aeruginosa*, their association with *bla*_KPC-2_ have recently been reported in various species from both clinical and environmental sources, suggesting that they have a broad host range and the ability to persist in the environment for long periods (Naas et al., 2013; Dai et al., 2016; Wang et al., 2017; Yao et al., 2017). Relatively few IncP-6 plasmids had been fully sequenced up to 2017, including Rms149 (AJ877225), pCOL-1 (KC609323), and p10265-KPC (KU578314) and pPAEC79 (CP040685.1) from clinical strains of *P. aeruginosa* (Haines et al., 2005; Naas et al., 2013; Dai et al., 2016; Wang et al., 2021); pRIO-5 (JF785550) from a clinical strain of *S. marcescens* (Bonnet et al., 2000; Bonnin et al., 2012); pRSB105 (DQ839391), a plasmid detected in a sample from a WWTP in Germany (Schlüter et al., 2007); and pHH2-227 (JN581942), an uncultured IncW/IncP-6 hybrid plasmid that was exogenously captured from an arable soil (Heuer et al., 2009; Król et al., 2013; Botts et al., 2017). Interestingly, only 16 unique plasmids containing both the IncP-6 replicon and the *bla*_KPC-2_ gene had been documented in the GenBank database until May 22^nd^ 2019 (Dong et al., 2020), while the number rises up to 25, according to plasmid database PLSDB by April 13^th^ 2021 (https://ccb-microbe.cs.uni-saarland.de/plsdb/) (Kukla et al., 2018; Galata et al., 2019).

It is of notice that two *bla*_GES-1/5_ IncP-6 plasmids have been recently published, pKRA-GES-5 (MN436715.1) found in clinical isolates of *K. pneumoniae* ST45 from Poland, and pN260-3 (AP023450) in a clinical *Enterobacter roggenkampii* co-harboring *bla*_IMP-1_ from Japan (Literacka et al., 2020; Umeda et al., 2021). Compared with IncP-6 archetype Rms149 and pWW14A-KPC, they had close to 100% of similarity with approximately 12.7-kb of the backbone containing partitioning, replication, and mobilization loci (Literacka et al., 2020), evidencing the remodeling ability of this platform.

### Genomic Comparison of the *bla*_KPC-2_ Genetic Environment From pWW14A-KPC2 and pWW19C-KPC2 With Those From Related Plasmids

The region harboring the *bla*_KPC-2_ gene in pWW14A-KPC2 is ~11 kb long and contains a Tn*3*-based transposon disrupted by an IS*Apu*-flanked element, where the core sequence is composed by ΔIS*Kpn6*/*bla*_KPC-2_-Δ*bla*_TEM-1_-IS*Kpn27*. This core module is connected with the gene cluster *korC-orfX-klcA-orfX-repB*. The resulting structure matched 99% to clinical isolates *C. freundii* M9169 and *E. cloacae* M11180 from Argentina (Gomez et al., 2011) and is associated with a truncated IS*Ec33* element. The resulting ΔIS*Ec33*-associated element is not bracketed by IRs and DRs, suggesting that its mobilization could be attributed to homologous recombination-based insertion of a foreign element Tn*3*-IS*Kpn27*-Δ*bla*_TEM-1_-*bla*_KPC-2_-IS*Kpn6*-*korC-orf-klcA-repB* into a pre-existent intact IS*Ec33* element (making it truncated at 3´ end), rather than resulting from a transposition event of the whole IS*Ec33*-associated element followed by the deletion of its adjacent extremities removing IR and DR sequences (Dai et al., 2016). This core platform was initially discovered in p10265-KPC. In the *bla*_KPC-2_ gene cluster of p10265-KPC, the primary genetic structure, Tn*3*-IS*Kpn27*-*bla*_KPC-2_-ΔIS*Kpn6*-*korC-orf-klcA-ΔrepB*, may have undergone two evolutionary events: (i) insertion of a *bla*_TEM-1_ gene between IS*Kpn27* and the Tn*3* IRR (right inverted repeat) and (ii) disruption of the *tnpA* gene (transposase) from Tn*3* by insertion of a composite transposon, IS*Apu1*-*orfX*-IS*Apu2* (Dong et al., 2020). Interestingly, pWW19C-KPC lacked ΔIS*Ec33* insertion sequence, indicating that the insertion of the *bla*_KPC-2_ cluster occurred at a different position in an IncP-6 backbone and seems to have a different evolutionary history of genetic assembly and transposition (**Figure 4**). The acquisition of the KPC-2 encoding region by IncP-6 replicons is in agreement with the results of previous studies, in which similar plasmids show remnants of multiple events, with intact or partial mobile elements dispersed throughout their sequences (Botts et al., 2017). Its dissemination could be an important contribution to the establishment of emerging clones as major nosocomial pathogens (Cejas et al., 2019).

## Conclusion

This is the first report on the genomic features of two non-clinical KPC-2-producing *Enterobacteriaceae* isolates from Argentina in terms of resistance determinants, genetic contexts of carbapenemase encoding genes, phylogeny, and virulence potential. To our knowledge, this is also the first report of IncP-6 plasmids circulating in Argentina and provides insights into the relevance of these plasmids in the maintenance and spread of KPC through the environment.

WW14A and WW19C had IncP-6 plasmids carrying *bla*_KPC-2_ in a Tn*3*-derived genetic element bearing a non-Tn*4401* structure and its full sequence was determined and compared with other IncP-6 plasmids, from diverse origins. The presence of a highly similar plasmid in different isolates from distant countries raises questions about mechanisms of persistence and dissemination and indicates it might play an important role in the horizontal dissemination of KPC-2 carbapenem resistance through wastewater and the spread from wastewater to humans and vice versa. Our findings underline the increasing importance of IncP-6 plasmids as environmental reservoirs and the spread potential of the resistance segments they carry through reshuffling with other plasmids.

None of the plasmids could be transferred by conjugation, due to the absence of the transfer system genes, but we hypothesize they could be mobilized by coresident plasmids present in both strains.

Given that *K. quasipenumoniae* and *Enterobacter* spp. are ubiquitous organisms isolated from a wide range of environmental niches and given the fact that *K. quasipneumoniae* may have been misidentified in clinical sources, being its clinical relevance underestimated, they might act as important vectors for the dissemination of plasmid-mediated carbapenem-resistance genes. Therefore, effective detection of such plasmids in carbapenem resistant isolates from wastewater may be used as a potential epidemiological indicator. Treatment methods in most WWTPs are usually not enough to mitigate resistance genetic determinants, therefore surveillance in sewage and urban effluents could provide monitoring data to understand the evolution of antimicrobial resistance in the environment. New strategies should also be developed to limit plasmid spread into bacterial populations.

## Data Availability Statement

The datasets presented in this study can be found in online repositories. The names of the repository/repositories and accession number(s) can be found below: https://www.ncbi.nlm.nih.gov/, PRJNA715927.

## Author Contributions

BG and MD, with the cooperation of GG and PPo conceived and designed the study. BG, MD, FB, PPe, GD’A, RF-E and LN performed experiments. BF, FE and NL performed WGS. BG, MD and MSH analyzed data. JC and RF-E provided assistance with MALDI-TOF/MS analysis and interpretation. MVH granted accession and supplied the water sample. MR provided laboratory infrastructure in Brazil. GG provided laboratory infrastructure in Argentina. BG, MD and MSH wrote this manuscript. All authors reviewed the manuscript. All authors contributed to the article and approved the submitted version.

## Funding

This work was supported by Agencia Nacional de Promoción Científica y Tecnológica PICT 2018-03413 to BG, and UBACyT 2018 - 20020170100473BA to GG.

## Conflict of Interest

Author MVH is employed by Agua y Saneamientos Argentinos S.A. (AySA), in Buenos Aires, Argentina.

The remaining authors declare that the research was conducted in the absence of any commercial or financial relationships that could be construed as a potential conflict of interest.

## Publisher’s Note

All claims expressed in this article are solely those of the authors and do not necessarily represent those of their affiliated organizations, or those of the publisher, the editors and the reviewers. Any product that may be evaluated in this article, or claim that may be made by its manufacturer, is not guaranteed or endorsed by the publisher.
