# Supplementary figures and images for: Characterization of Emerging Pathogens Carrying bla KPC-2 Gene in IncP-6 Plasmids Isolated From Urban Sewage in Argentina
Source: Front Cell Infect Microbiol. 2021 Aug 24;11:722536. doi: 10.3389/fcimb.2021.722536 (PMC8421773; doi:10.3389/fcimb.2021.722536)

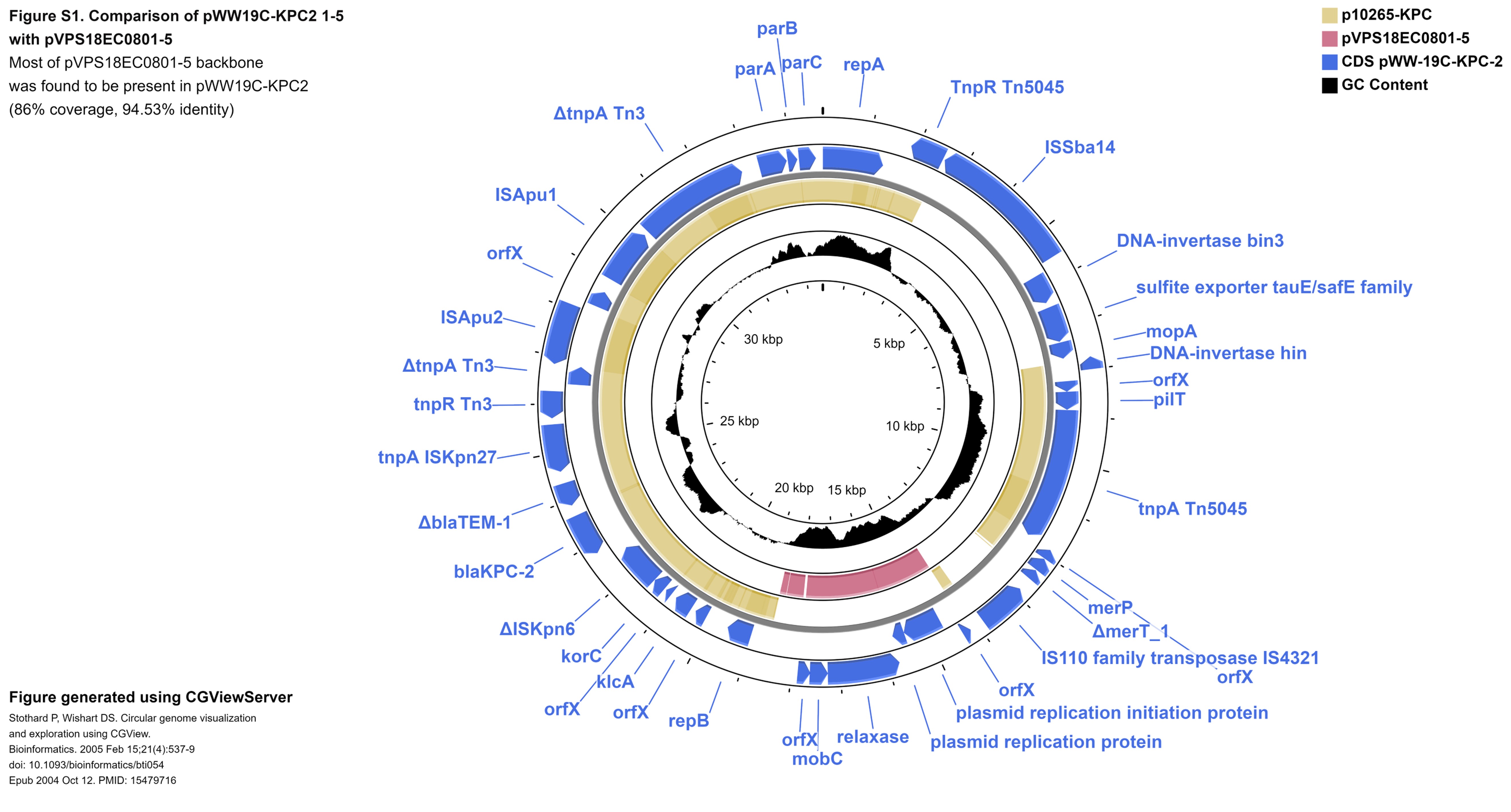

Supplement: Supplementary file 1 [file Image_1.jpeg]
