# Supplementary material for: Characterization of Emerging Pathogens Carrying bla KPC-2 Gene in IncP-6 Plasmids Isolated From Urban Sewage in Argentina
Source: Front Cell Infect Microbiol. 2021 Aug 24;11:722536. doi: 10.3389/fcimb.2021.722536 (PMC8421773; doi:10.3389/fcimb.2021.722536)
Supplement: Supplementary file 3 [file Table_2.docx]

**Table S2.** Diverse origin plasmids sharing pWW-19C-KPC2's 5.2 kb extra region

| Plasmid | Accession no. | Inc group | Origin | Total length (bp) | % Query Coverage | Percent Identity |
| --- | --- | --- | --- | --- | --- | --- |
| *Klebsiella pneumoniae* strain KP2611 plasmid pKP2611-N, complete sequence | MN967025 | IncN2 | liver abscess, Taiwan | 60970 | 100% | 99.96 |
| *Klebsiella pneumoniae* strain 130002 plasmid pKPC2_130002, complete sequence | CP064852 | IncFII | blood, China | 94968 | 100% | 99.94 |
| *Klebsiella pneumoniae* strain 3214 plasmid pNDM_3214, complete sequence | CP028851 | Non-typable | Sputum, China | 238037 | 99% | 99.94 |
| *Klebsiella* sp. WP8-S18-ESBL-06 plasmid pWP8-S18-ESBL-06_1 DNA, complete genome, strain: WP8-S18-ESBL-06 | AP022257.1 | IncFIB(K) | wastewater treatment plant effluent, Japan | 128811 | 99% | 99.9 |
